# Characterization of reward and effort mechanisms in apathy

**DOI:** 10.1016/j.jphysparis.2014.04.002

**Published:** 2015

**Authors:** Valerie Bonnelle, Kai-Riin Veromann, Stephanie Burnett Heyes, Elena Lo Sterzo, Sanjay Manohar, Masud Husain

**Affiliations:** a Dept. of Experimental Psychology, University of Oxford, Oxford, UK; b Institute of Cognitive Neuroscience, University College London, UK; c Nuffield Dept. Clinical Neurosciences, University of Oxford, Oxford, UK

**Keywords:** LARS-e, Lille Apathy Rating Scale – extended version, AI, Action Initiation, ER, Emotional Responsiveness, IC, Intellectual Curiosity, SA, Self-Awareness, DASS, Depression, Anxiety, Stress Scales, SHAPS, Snaith–Hamilton Pleasure Scale, MVC, Maximum Voluntary Contraction, IP, Indifference Point, IL, Indifference Line, AIC, Akaike Information Criteria, PD, Parkinson’s disease, Motivation, Decision-making, Action Initiation, Effort discounting, Apathy

## Abstract

Apathy is a common but poorly understood condition with a wide societal impact observed in several brain disorders as well as, to some extent, in the normal population. Hence the need for better characterization of the underlying mechanisms. The processes by which individuals decide to attribute physical effort to obtain rewards might be particularly relevant to relate to apathy traits. Here, we designed two paradigms to assess individual differences in physical effort production and effort-based decision-making and their relation to apathy in healthy people. Apathy scores were measured using a modified version of the Lille Apathy Rating Scale, suitable for use in a non-clinical population.

In the first study, apathy scores were correlated with the degree to which stake (reward on offer) and difficulty level impacts on physical effort production. Individuals with relatively high apathy traits showed an increased modulation of effort while more motivated individuals generally exerted greater force across different levels of stake. To clarify the underlying mechanisms for this behavior, we designed a second task that allows independent titration of stake and effort levels for which subjects are willing to engage in an effortful response to obtain a reward. Our results suggest that apathy traits in the normal population are related to the way reward *subjectively* affects the estimation of effort costs, and more particularly manifest as decreased willingness to exert effort when rewards are small, or below threshold. The tasks we introduce here may provide useful tools to further investigate apathy in clinical populations.

## Highlights

• Apathy in the normal population is dissociable from depression and anhedonia.
• Apathy in the normal population is related to the modulation of physical effort people are willing to engage.
• Apathy in the normal population is associated with higher *subjective* effort costs for small rewards.

## 1 General introduction

Apathy has been defined as a loss of motivation characterized by a quantitative reduction of self-generated voluntary and purposeful behaviors (Levy and Dubois, 2006). It is frequently observed across several neurological and psychiatric conditions, including Parkinson’s disease (Pedersen et al., 2009), stroke (Caeiro et al., 2013), Alzheimer’s disease (Robert et al., 2010), traumatic brain injury (Marin and Wilkosz, 2005), major depressive disorder (Treadway and Zald, 2011) and schizophrenia (Foussias et al., in press). Besides being common in many different clinical populations, apathy is now also recognized to be widespread in milder form in healthy people, particularly in the elderly (Brodaty et al., 2010; Clarke et al., 2010). But even in young people, a motivation is an important factor that contributes to lack of success in gaining employment (Vansteenkiste et al., 2004, 2005). Apathy therefore has wide societal impact, both in health and disease.

Despite being a common problem, apathy is an under-recognized and relatively poorly understood syndrome. Although mechanisms responsible for apathy remain to be established, three subtypes of disrupted processing have been proposed: emotional, cognitive and behavioral (Stuss et al., 2000). *Emotional apathy* is characterized by a lack of feeling, emotion or concern and a blunting of personality. *Cognitive apathy* relates to impaired elaboration of plans for action. *Behavioral apathy*, also termed ‘auto-activation deficit’ by some authors, refers to diminished self-initiated actions, lack of effort, decrease in productivity, or more generally a quantitative reduction in self-generated voluntary behaviors (Marin and Wilkosz, 2005). It often manifests as physical inertia, with requirement for prompts to initiate physical activity. In this article, we focus on this particular domain of apathy.

Although very few experimental studies have been conducted to probe underlying mechanisms, some recent investigations have demonstrated that insensitivity to rewards, abnormalities in estimating the cost of effortful behavior, or disconnection of reward evaluation from motor output, might be important components of behavioral apathy in patients with focal lesions, e.g. stroke affecting the basal ganglia (Schmidt et al., 2008; Adam et al., 2013; Rochat et al., 2013), but also in patients with psychiatric disorders (Gold et al., 2013; Treadway et al., 2012).

There is a general agreement that humans tend to avoid effortful actions and, when given a choice, display a preference for strategies that minimize effort (e.g. Friman and Poling, 1995; Kool et al., 2010; Walton et al., 2006). Thus, when deciding whether to take a particular course of action or not, people weigh the value of prospective rewards against the amount of effort that is required to attain them (Bijleveld et al., 2012). Rewards which require less effort to obtain are preferred over the same value rewards requiring greater effort for receipt. That is, behavior is driven by the net-value (benefits minus costs) of rewards (Bijleveld et al., 2012; Botvinick et al., 2009; Kivetz, 2003). This has been referred to as *effort discounting.*

As far as we are aware, this principle has not been investigated specifically in the context of apathy, but some researchers have proposed that excessive effort discounting might be a key component of this condition (Kurniawan et al., 2010). That is, individuals with behavioral apathy might be highly sensitive to action costs, assessing many activities to be too effortful and thus not worth performing or engaging in. Therefore, studies that investigate cost-benefit decision-making have the potential to provide valuable insights into mechanisms underlying behavioral apathy.

There have been several methodological developments in terms of how physical effort discounting is assessed (e.g. Gold et al., 2013; Kurniawan et al., 2010; Treadway et al., 2009). For instance, Pessiglione and colleagues (Schmidt et al., 2008) developed a paradigm where participants are presented with different monetary incentives (stakes) and have to respond by squeezing a hand grip, knowing that the proportion of the monetary stake they will obtain depends upon the force they exert. On this task, patients with apathy following lesions of the basal ganglia showed less response force modulation in response to the different incentives offered, but preserved valuation modulation (increased ‘liking’ from low to high incentives as measured by skin conductance) (Schmidt et al., 2008).

In the first study presented here, we used a similar task with additional manipulations to further investigate how effort exertion is modulated in response to an incentive but also to other external factors such as the level of difficulty and visual feedback of response. These additions allow investigation of other important aspects of apathy, namely sensitivity to effort requirement and Self-Awareness of performance via feedback. Our objective was to examine how these manipulations affect motivation, and more particularly, how they vary in relation to apathy traits in the healthy population.

**Fig. 1:**
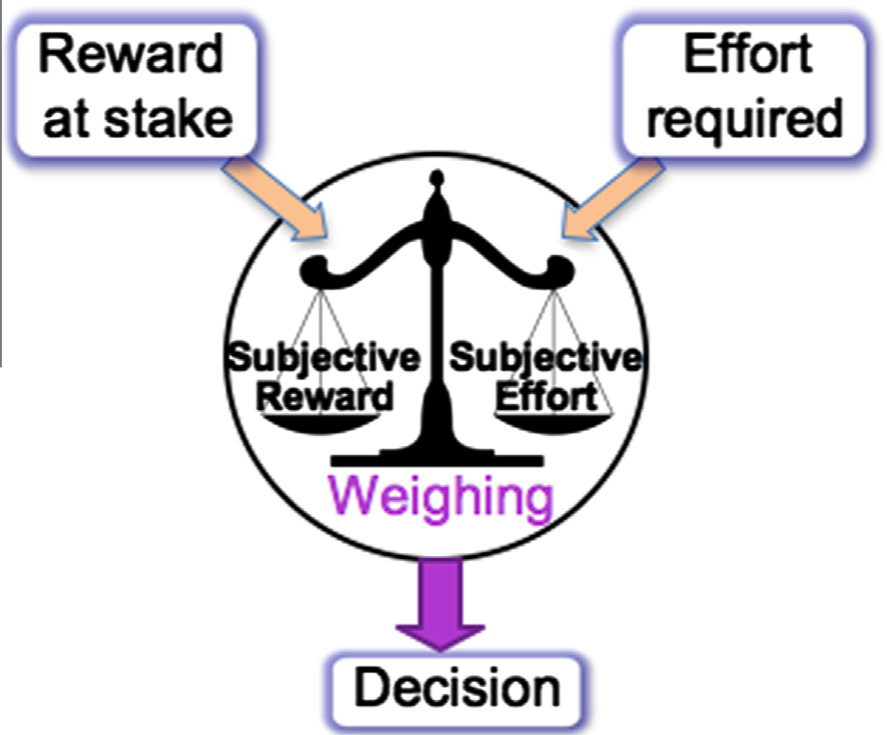
Schematic representation of effort and reward-based decision-making. Objective external information on reward level offered and effort requirement are weighed against each other to produce subjective values, which vary across individuals. The output of this weighing process leads to the decision about whether the reward is worth the effort cost, or not.

Although this approach constitutes a very useful way to investigate aspects of physical effort production in relation to apathy, there might be other means by which to explore underlying decision-making mechanisms. At least three processes might be involved before the decision to exert effort for a reward is made (Fig. 1):

(1) *Reward sensitivity:* how motivation is modulated by a change of reward, regardless of effort requirement.
(2) *Effort sensitivity:* how motivation is affected by an increase in effort requirement, regardless of reward at stake.
(3) *Subjective effort discounting:* how effort costs are weighed against reward value to compute a subjective value for a given action.

We therefore designed a second task to investigate how these three processes might relate to *behavioral apathy* across healthy individuals. This task allowed titration, for each individual, of an ‘Indifference Point’ (IP) for each reward magnitude – the effort which an individual would be willing to make to obtain the reward on 50% of occasions when offered that reward. A similar approach has been used in the temporal discounting literature to relate the characteristics of indifference functions to impulsivity traits (Kable and Glimcher, 2007). With this design, we were able to investigate how participants’ choices to engage in an effortful response are influenced by rewards at stake (reward sensitivity) or effort requirements (effort sensitivity). In addition, we were able to characterize the way effort cost is subjectively weighed against reward, with the estimation of IPs.

Specific hypotheses and methods for each of the studies outlined are presented in Sections 3 and 4 respectively, after the description of the Methods that are common to both.

## 2 Methods

### 2.1 Participants

The study was approved by Oxford University Medical Sciences Inter Divisional Research Ethic Committee. All subjects volunteered for the study via a website and gave informed written consent before the study. Fifty neurologically healthy participants, with no current diagnosis of psychiatric disorder were recruited for study 1, and thirty were recruited for study 2. The two studies lasted 90 min. Participants were told that the money they would receive at the end of the experiment would depend on their task performance and would vary between £8 and 12.

### 2.2 Questionnaires

#### 2.2.1 Apathy questionnaire

The Lille Apathy Rating Scale (LARS) (Sockeel et al., 2006) is a semi-structured interview assessing clinically relevant levels of apathy along several domains reflecting the distinct components of apathy (emotional, cognitive and behavioral). Concurrent validity has been assessed in relation to the Apathy Evaluation Scale (Marin et al., 1991), with correlations of *r* = 0.87 between global scores suggesting high concurrent validity, and with reference to expert clinician categorization of syndrome severity. Moreover, the LARS has been shown to assess apathy symptoms independently of depression (Sockeel et al., 2006).

However, some of the questions would not be appropriate for healthy participants. We therefore modified the original questionnaire to produce an extended version (LARS-e), a 51-item questionnaire, which like the original LARS, assesses four domains of apathy, with each domain consisting of subscales relevant to distinct features of apathy:

• **Action Initiation (AI)** comprises two subscales, everyday productivity and initiative. It mostly refers to physical aspects of apathy and can therefore be used as an index of *behavioral apathy*.
• **Intellectual Curiosity (IC)** comprises four subscales, interest, novelty seeking, motivation and social life. It mostly refers to intellectual aspects of apathy and can be used as an index of cognitive apathy.
• **Emotional Responsiveness (ER)** has only one subscale that can be used as an index of emotional apathy.
• Apathy is often associated with low *Self-Awareness* (*SA*), it is therefore a relevant additional axis to index.

A copy of the LARS-e questionnaire can be viewed in Supplementary material.

Here, we used the combined LARS-e scores as a general index of apathy traits (with low scores associated with greater apathy), and the AI domain scores as a specific index of *behavioral apathy*.

In adapting the LARS to create the LARS-e we were motivated to retain the original apathy construct but to increase sensitivity to subtle differences in levels of apathy in moderately impaired and healthy populations. In the current study, participants provided self-report responses.

The original yes/no response format was replaced with a four-response option Likert scale (1–4) in order to extend the operating range of the instrument, 1 corresponding to extremely apathetic and 5 extremely motivated. In a pilot study, we found that overall scores on the LARS-e were correlated (*r* = 0.654, *p* < 0.01) with scores on the Apathy Scale (Starkstein et al., 1992); for all subscales except Self-Awareness, subscale scores on the original LARS and LARS-e were also significantly correlated (*r* > 0.4, *p* < 0.05). All subscales apart from the Self-Awareness one had a reasonable index of reliability (see Supplementary information for more details).

#### 2.2.2 Depression questionnaire

The Depression, Anxiety, Stress Scales (DASS) (Lovibond and Lovibond, 1995), a questionnaire developed in a non-clinical population, was used to measure depression. The questionnaire consists of 42 items divided into three subscales assessing depression, anxiety, or stress. Each of the three subscales contains 14 items that are scored on a four-point Likert scale.

#### 2.2.3 Anhedonia questionnaire

The Snaith–Hamilton Pleasure Scale (Snaith et al., 1995) was used to measure anhedonia. This 14-item questionnaire is scored on a four-point scale.

A factor analysis was performed to investigate whether apathy scores relate to depression or anhedonia (see SI Section 1.2 and Table S1). To summarize the main findings, while the three other components of the LARS-e appear partially related to anhedonia, AI does not. As we discuss later, this might be an important dissociation to bear in mind when examining components of apathy and/or anhedonia.

### 2.3 Apparatus

**Fig. 2:**
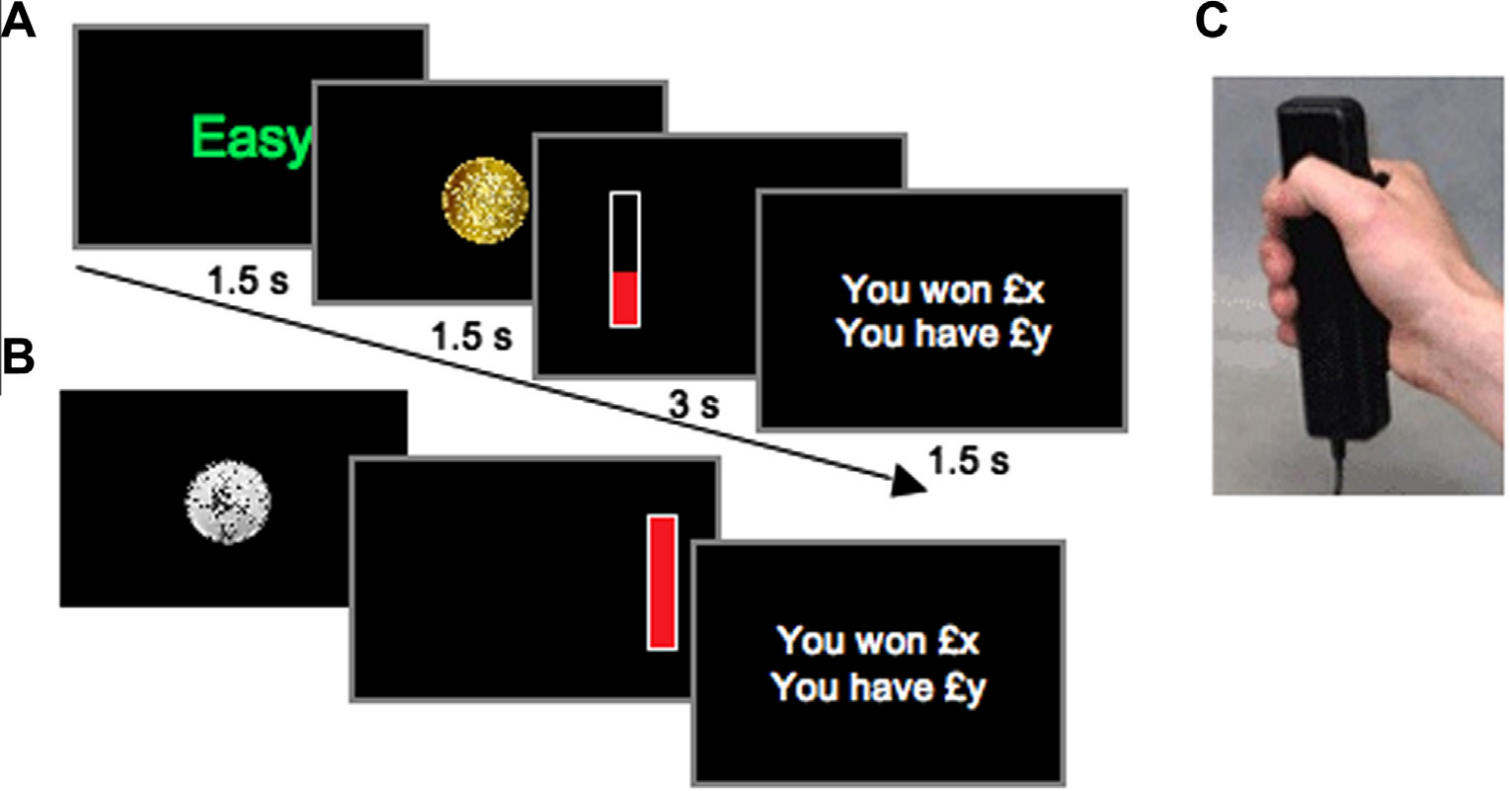
Experimental task used in Study 1. Participants were presented with a monetary reward cue (£1 or 10 pence – £0.1 – coin) and on two third of trials a difficulty level cue (Easy or Difficult) was presented. (A) Trial in which difficulty cue is presented with visual feedback of response (level of red bar). (B) Trial where only stake value is presented with no visual feedback of response (red bar full throughout). The order of stake and difficulty cues was counterbalanced across trials. The response period started with the presentation of a vertical bar on one side of the screen and participants had to squeeze the corresponding handgrip (C) to win a percentage of the money at stake. On half of the trials, response feedback was provided, in the form of a red level indicating the percentage of the stake that can be won with the current force exerted.

Stimulus presentation was programmed in MATLAB (The MathWorks Inc., USA) using the Psychtoolbox (http://psychtoolbox.org). Force was recorded using a TSD121B-MRI hand dynamometer (BIOPAC Systems Inc., USA) with a sample rate of 500 Hz (Fig. 2C). The recorded signal was digitalized and fed in real-time into the PC running the task program.

### 2.4 Estimation of Maximal Voluntary Contraction (MVC)

The two tasks were calibrated using individuals’ Maximum Voluntary Contraction (MVC). At the beginning of the experimental session, participants were asked to squeeze the handles as strongly as they could with their right and left hands. The maximum force was recorded for each hand, and participants were then instructed to try to squeeze each handle until the level indicating their force online went above a yellow bar, corresponding to 100% of the maximum force previously recorded for the same hand. If they managed to reach that level, the procedure was repeated using the new maximum, else the first value was kept. The procedure was repeated three times and the maximal force reached was used as the MVC for that participant.

## 3 Study 1: modulation of effort production by stake, difficulty and response feedback

### 3.1 Objectives and hypotheses

The aim of Study 1 was to examine how three factors – reward magnitude, effort requirement, and the presence of performance feedback (online visual feedback of the amount of force produced) – relate to apathy traits in a sample of the healthy population. For this, we developed an experimental task adapted from Schmidt et al. (2008), which required participants to effortfully squeeze a handgrip in order to win monetary incentives. We hypothesized that high apathy traits would be associated with less intrinsic motivation and more behavioral inertia, which would in turn manifest as lower response force exertion, especially under more challenging conditions such as high difficulty level or absence of visual feedback, and/or lower motivation for rewards, perhaps due to a higher reward sensitivity threshold.

### 3.2 Methods

#### 3.2.1 Participants

Fifty neurologically healthy participants (18 males, 32 females) aged 18–65 (mean age 29.5 ± 12.6) were recruited for this study.

#### 3.2.2 Task description

The experimental task manipulated *stake magnitude*, *difficulty level* and online visual presentation of the force applied during the response, or ‘*response feedback*’ (Fig. 2). A total of 288 trials were grouped into six blocks, each lasting approximately five minutes. Between each block, participants were given time to rest or fill in the questionnaires (LARS-e, DASS and SHAPS). The money participants received at the end of the experiment was equal to the cumulative total won in one of the six blocks (selected by chance with a dice), and was typically between £8 and 12.

Each trial began with the presentation of either a monetary incentive or *level of stake* (10 pence (£0.1) or £1 coin) or a *difficulty level* cue (easy, difficult or no cue) indicating how hard it would be to win a percentage of the monetary stake (Fig. 2A). The formula for computing reward was the following:

Reward=Reward Gain×Stake×(Response Force/MVC)

where reward gain was 0.7 for easy trials and 0.3 for difficult trials and MVC is the Maximum Voluntary Contraction (see Section 2.4 above).

The order of the stake and difficulty cues was counterbalanced across trials. Each cue was presented for 1.5 s.

On one third of the trials, only the monetary incentive was shown (Fig. 2B). These trials still had different difficulty levels, but participants could only find out the trial difficulty level while responding (if response feedback was on) or at the end of the trial during the reward feedback period (if response feedback was off). After cue presentation, the response period started with the appearance of a vertical bar on the left or the right of the screen, prompting participants to start responding with the corresponding hand (Fig. 2A and B). The response period lasted 3 s. The harder the participants squeezed the hand dynamometer (Fig. 2C), the more money they earned.

In trials where visual response feedback was provided (Response feedback ‘ON’), the vertical bar filled in red according to the force exerted. If participants exerted their Maximal Voluntary Contraction (MVC), the red level would rise up to 70% of the box on easy trials, or 30% on difficult trials. In trials in which visual response feedback was not provided (Response feedback ‘OFF’), the bar appeared immediately entirely filled in red (Fig. 2B). At the end of the trial, participants were shown how much money they won in that particular trial and how much they had gained in total.

### 3.3 Results

#### 3.3.1 Stake, difficulty and response feedback influence effort production

A 2 × 2 × 2 ANOVA was used to analyze the effects of stake (£0.1 or £1), difficulty (easy or difficult) and response feedback (‘ON’ or ‘OFF’) on response force. Participants exerted significantly more effort on trials when £1 was at stake, compared to trials where maximum reward was £0.1 (*F*(1, 49) = 34.17, *p* < .001). In addition, they produced significantly higher response forces on easy trials, compared to difficult trials (*F*(1, 49) = 14.41, *p* < .001). Participants also exerted more effort when they could see online visual feedback (Response feedback ‘ON’) of their response force compared to trials where no feedback was provided (*F*(1, 49) = 111.68, *p* < 0.0005).

**Fig. 3:**
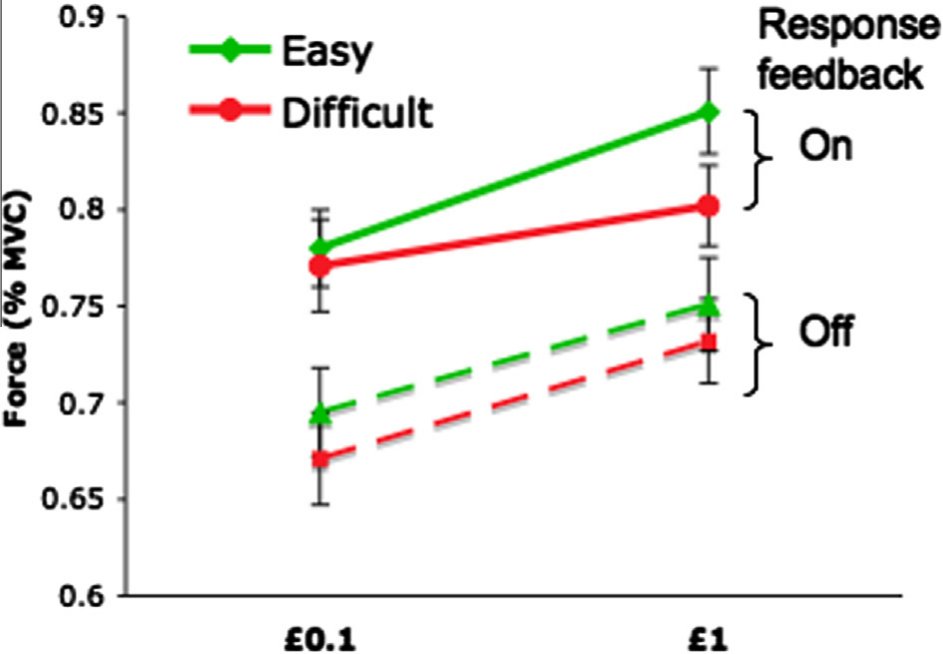
Effect of stake, difficulty and response feedback on force exerted (index of motivation). Average response force, expressed as percentage of Maximum Voluntary Contraction (MVC) are plotted for the different stakes (£0.1 and £1) and the different difficulty levels (easy in green, difficult in red), for trials were response feedback was provided (solid lines) or not (dotted lines). Error bars represent standard errors.

There was a significant interaction between stake and difficulty cues (*F*(1, 49) = 8.501, *p* = 0.005), driven by the difficulty cue producing more effort modulation for the higher stake (Fig. 3). Thus participants were more motivated to exert a greater force for higher stakes when the trial was an easy one. Additional effects related to the difficulty cues are presented in Supplementary Materials.

In addition, there was a significant interaction between the three task manipulations (stake × difficulty level × response feedback, *F*(1,49) = 11.52, *p* = 0.001). This was driven by the fact that the stake × difficulty interaction mentioned above was observed only when the response feedback was on (Fig. 3), suggesting that during the response phase feedback amplifies the motivational impact of the cues (presented prior to response).

#### 3.3.2 Relationship with apathy traits

The self-rating scores obtained with the LARS-e (see Section 2) were used to correlate apathy traits with behavioral effects on the task. The combined LARS-e scores were used as an index of general apathy traits (low scores mean more apathy), and the AI domain scores as a specific index of behavioral apathy.

##### 3.3.2.1 Overall response force

**Fig. 4:**
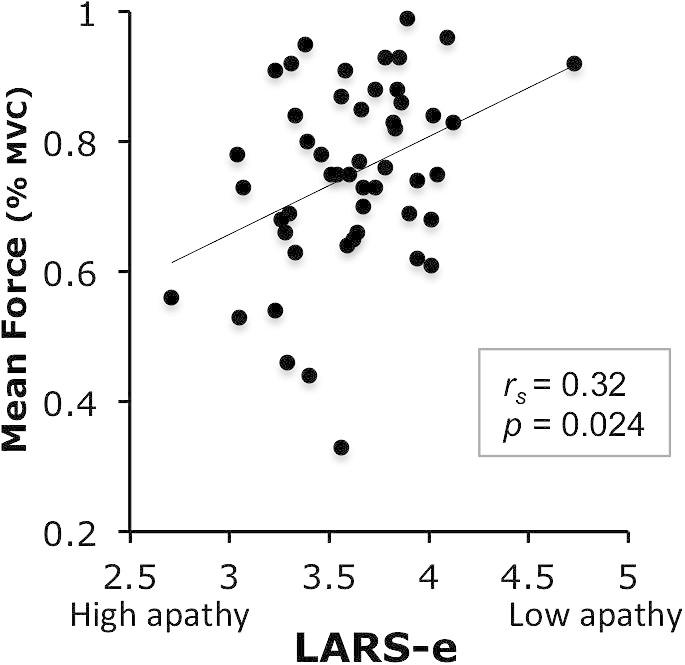
Relation between LARS-e scores and task performance. Relationship between LARS-e scores (*higher scores* = *more motivated individuals*) and mean of overall response force, expressed as percentage of MVC, across all trials.

As hypothesized, subjects with higher apathy traits (lower LARS-e scores) exerted significantly less effort on average, as demonstrated by a positive correlation between the overall LARS-e score and mean response force across all trials (spearman *r_s_* = 0.32, *p* = 0.024) (Fig. 4). A partial correlation controlling for depression and anhedonia confirmed the specificity of the relationship between LARS-e scores and overall response force (*r* = 0.393, *p* = 0.008), so that individuals who are in general more apathetic respond overall less strongly.

##### 3.3.2.2 Modulation of effort with incentive (stake) and difficulty

We next investigated in more detail the relation between apathy traits and the effects of stake and difficulty cues on *modulation of effort*. Overall LARS-e scores were significantly negatively correlated with the *Stake effect* – defined as percentage change in force exerted for £1 vs. for £0.1 (*r_s_* = −0.302, *p* = 0.033). Thus, more apathetic people (low LARS-e scores) showed greater percentage increase in force for £1 vs. £0.1 than more motivated individuals. LARS-e scores were also significantly negatively correlated with the *Difficulty effect* – defined as percentage change in force exerted for ‘Easy’ vs. for ‘Difficult’ trials (*r_s_* = −0.316, *p* = .031). More apathetic people showed greater percentage increase in force on ‘Easy’ compared to ‘Difficult’ trials than more motivated individuals. Both relationships remained significant after controlling for depression or anhedonia, using partial correlations (Stake effect: *r* = −0.344, *p* = 0.024; Difficulty effect: *r* = −0.354, *p* = 0.020). These findings show that individuals with higher apathy traits (lower LARS-e scores) modulated their response force *more* according to cues related to reward and difficulty level relative to more motivated participants.

**Fig. 5:**
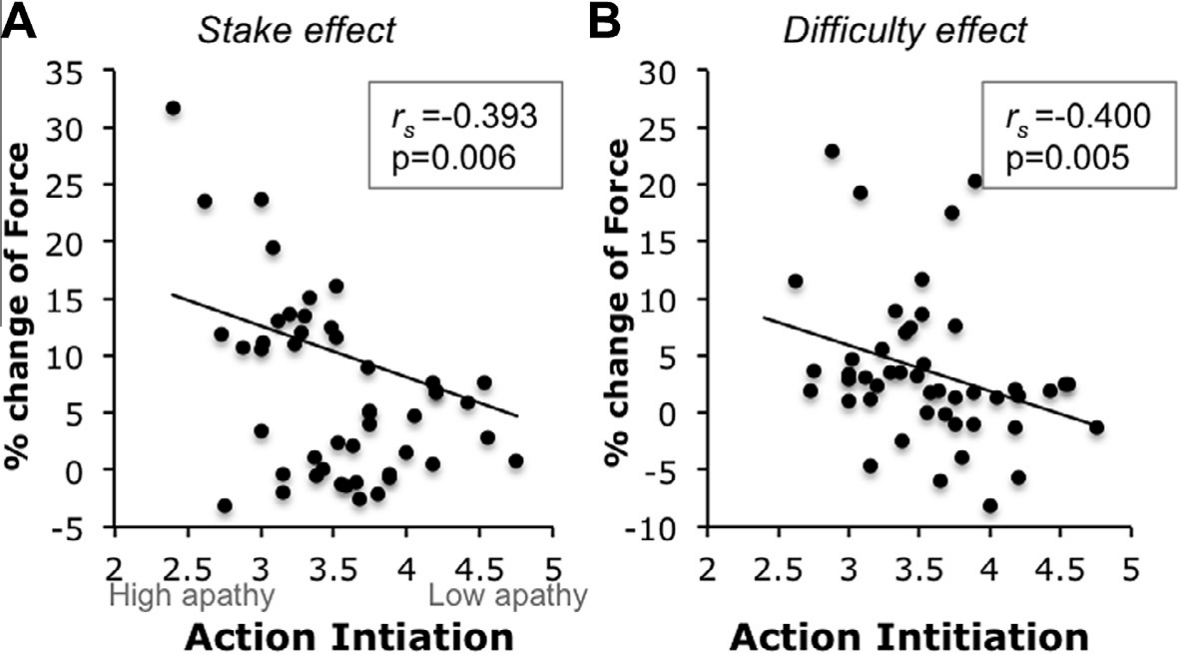
Relation between behavioral apathy and task performance. (A) Correlation between Action Initiation subscale of the LARS-e and effect of stake on change of response force (percentage change from £0.1 to £1) and (B) effect of difficulty on change of response force (percentage change from difficult to easy trials).

To investigate more specifically the relation with behavioral apathy traits, we examined the relationships between stake and Difficulty effect and the AI subscale of the LARS-e. AI scores correlated with the stake effect (*r_s_* = −0.296, *p* = 0.037, Fig. 5A), and the Difficulty effect (*r_s_* = −0.400, *p* = 0.005) (Fig. 5B), suggesting that, although the *overall response force* does not seem to be related to behavioral apathy traits, the *modulation of response force* is.

As for the overall LARS-e scores, both correlations remained significant when controlling for depression and anhedonia (Stake effect: *r* = −0.393, *p* = 0.006; Difficulty effect: *r* = −0.442, *p* = 0.005). In addition, these effects were not confounded by other apathy domains. Indeed, partial correlations with AI controlling for ER, IC and SA scores remained significant (Stake effect: *r* = −0.310, *p* = 0.043; Difficulty effect: *r* = −0.400, *p* = 0.008).

**Fig. 6:**
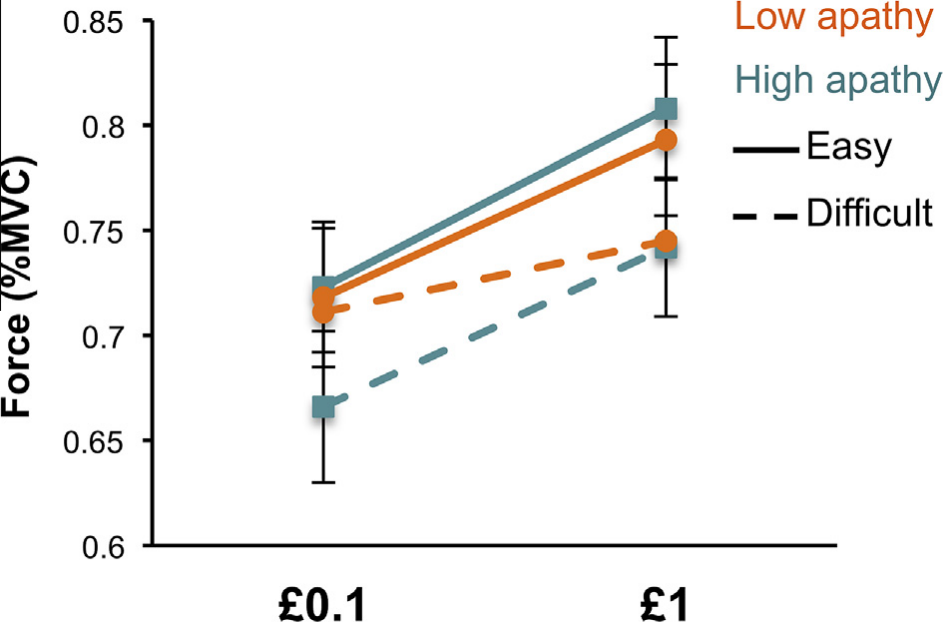
High and low behavioral apathy group comparison Average response force, expressed as percentage of Maximum Voluntary Contraction (MVC) are plotted for the different stakes (£0.1 and £1) and the different difficulty levels (easy: solid line, difficult: dotted line), for participants with high (orange) and low (cyan) Action Initiation scores (lower scores mean more behavioral apathy). Error bars represent standard errors.

To further investigate the relation between behavioral apathy traits and the changes in motivation associated with rewards at stake or difficulty level, we split participants into two groups with high and low AI scores using a median split and performed a group × stake × difficulty ANOVA. There was a significant interaction between group × stake × difficulty (*F* = 3.97, *p* = 0.05) (Fig. 6), which was driven by the fact that the high apathy group responded less vigorously for the least motivating condition (low stake, high difficulty).

More details on the relationship between apathy traits and the difficulty cues effects are presented in Supplementary Information.

##### 3.3.2.3 Modulation of effort with response feedback

There was no significant correlation between LARS-e (*r* = −0.199, *p* = 0.166) or AI scores (*p* = 0.941) and modulation of effort production by response feedback.

Importantly, none of the behavioral effects nor the overall response force correlated with depression (overall response: *p* > 0.9, Stake effect: *p* > 0.8, Difficulty effect: *p* > 0.8, Feedback effect: *p* = 0.15) or anhedonia (overall response: *p* > 0.6, Stake effect: *p* > 0.5, Difficulty effect: *p* > 0.5, Feedback effect: *p* = 0.19) questionnaires scores, confirming that the experimental task was purely related to apathy and not to depression or anhedonia.

### 3.4 Conclusion

The results of this study demonstrated that motivation (here indexed by willingness to exert physical force) can be up-regulated by increasing the reward at stake *or* decreasing the level of difficulty to be expected (Fig. 3). In addition, motivation can be decreased during the response execution period, by not providing on-line visual feedback of the force exerted (Fig. 3). Importantly, individual differences in some of these behavioral effects are related to self-reported apathetic traits (Figs. 4 and 5) as indexed using the LARS-e. Individuals with more pronounced *behavioral apathy* traits were *more* sensitive to reward and difficulty, but not to response feedback (Figs. 4 and 5), as shown by a steeper modulation or response force from one condition to the other.

Three distinct processes could account for these observations:

(i) Increased *motivational threshold for reward*: behavioral apathy might be associated with having a higher threshold for reward magnitude that is considered worth making an effortful response for, resulting in weaker responses for low rewards but similar responses for high enough reward.
(ii) Increased *effort sensitivity*: While all people are generally disposed to avoid effortful actions (e.g. Friman and Poling, 1995; Kurniawan et al., 2011), apathetic individuals’ low willingness to incur costs might in part be driven by placing abnormally large emphasis on action costs.
(iii) Change in activation threshold after reward and effort costs have been *subjectively* combined to guide behavior: apathetic people might rely on an aberrant cost-benefit valuation system.

As a next step, we designed a study that permits investigation of these processes, to investigate which of these relate to apathy traits.

## 4 Study 2: sensitivity to reward and effort, and effort discounting

### 4.1 Objectives

In Study 1, apathy traits were found to be related to the level of physical effort individuals have to exert in more difficult, and less rewarding conditions. However, it is unclear whether this is due to a difference in sensitivity to stake or effort, or alternatively in the way these two factors influence each other, e.g. in the way stake is subjectively devalued under more effortful conditions (Fig. 1). Therefore, we next investigated more specifically the processes underlying effort-based decision making, using a new task that allows us to define more precisely under what circumstances a subject decides an action is no longer worth pursuing.

In this experiment, participants are offered, on a trial-by-trial basis, different magnitudes of stake for different levels of effort. Using an adaptive staircase procedure we were able to compute, for each subject, individual ‘Indifference Points’ (IP) – the point at which participants decide on 50% of occasions that they are willing to exert a certain force (as a percentage of MVC) for a particular stake. Our modeling of choices provided an estimate of participants’ sensitivity to reward and effort, as well as measures of the characteristics of subject-specific effort-cost functions. We then examined how these measures related to individual differences in apathy traits.

### 4.2 Methods

#### 4.2.1 Participants

Thirty participants were recruited for this study via a local advertising website (mean age 25.3 ± 4.2, range 18–34, 13 males).

#### 4.2.2 Task description

This paradigm focused on the effort and reward based decision-making process taking place *before* engaging in a physically effortful response. As with the previous task, we wanted to keep the cognitive demands to a minimum, so that it would not be a confounding factor in our analysis of the relationship between task behavior and apathy traits. We also tried to make the task more engaging and easy to translate into a ‘real-life’ situation.

**Fig. 7:**
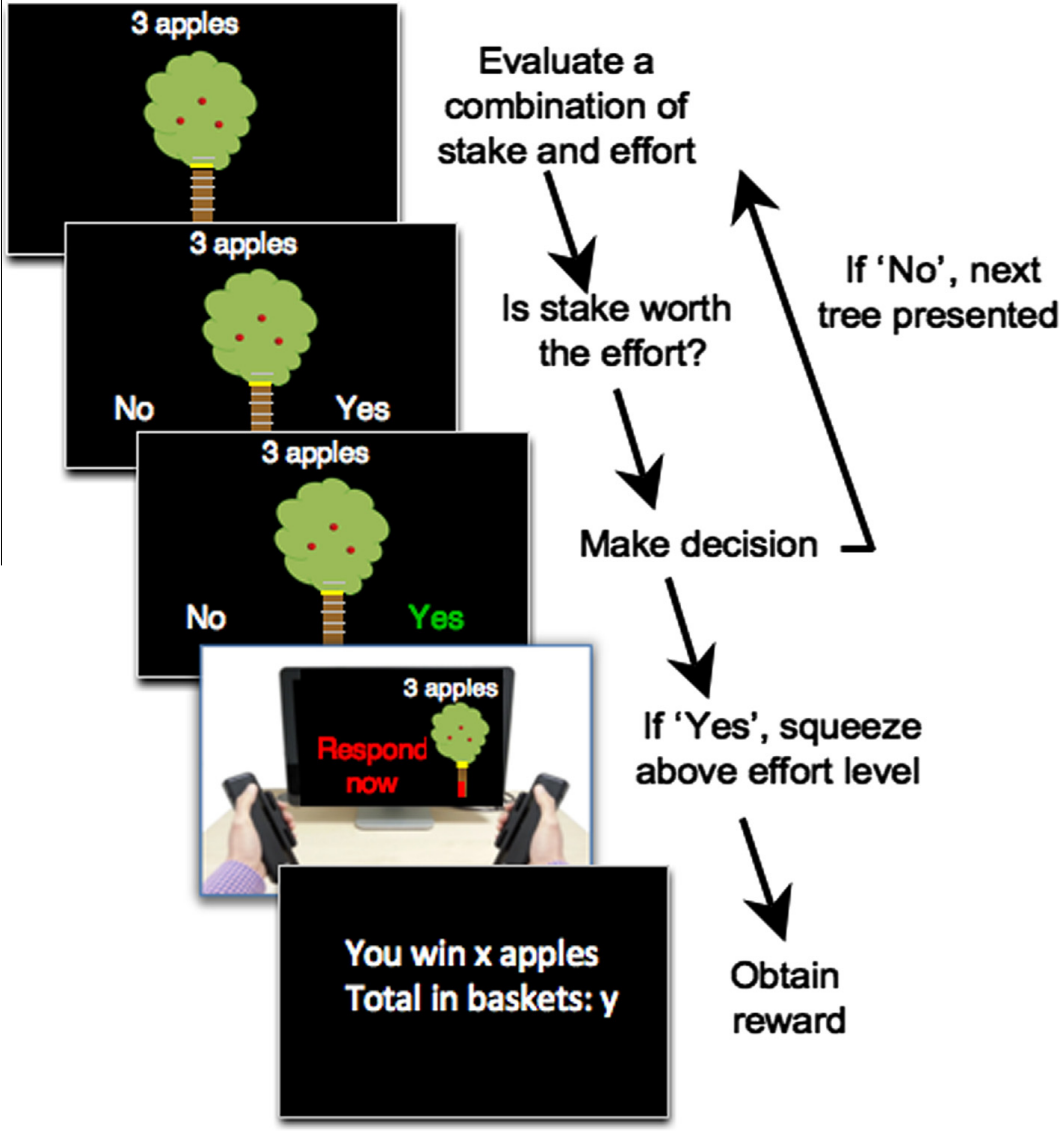
Adaptive effort discounting task. Trials start with the presentation of an apple tree for 1.5 s. The number of apples in the tree represents the stake while the effort level is indicated by the trunk size and the level highlighted in yellow on the scale. This is followed by the presentation of the yes/no option at the bottom of the screen. Subjects can select the option corresponding to their choice by gently squeezing the correct response device. If the yes option is selected, the same tree reappears on the right or the left of the screen and participants have 3 s to squeeze using the corresponding hand. Subjects only win a percentage of the stake if they manage to reach or go beyond the top of the trunk. If the no option is selected, the next trial starts with the presentation of another tree (i.e. another offer with a different combination of stake and effort required to obtain it).

Each trial started with the presentation of an apple tree that combined information about the stake (number of apples) and the effort level required to win a fraction of this stake (trunk height) (Fig. 7). There were six possible stakes (1, 3 6, 9, 12 and 15 apples), and six possible effort levels (60%, 70%, 80%, 90%, 100%, 110% of subject’s MVC), indicated by yellow horizontal lines on the tree trunk. The highest level (110% MVC) was of course not reachable, but was added nonetheless to control the number of effortful responses in the case some individuals would always agree to respond to the 100% MVC effort level trials (see staircase procedure below). Trials where the effort level was 110% of the MVC were not included in the analysis.

At the beginning of the experiment, participants were trained on these different levels so that they knew the physical effort level each yellow line corresponded to when they started the task. In addition, although participants were not explicitly told so, the first block was considered as training and not included in the analysis. Direct visual feedback of the amount of effort exerted in relation to the level required to obtain the reward was provided by a red bar that filled the trunk as participants squeezed the handgrip device (see Fig. 7).

On each trial, participants had to decide whether or not they wanted to engage in an effortful response to win a percentage of the presented stake. They selected a ‘yes’ or ‘no’ option by gently squeezing one of the handle corresponding to the location of the option on the screen. If the combination [stake/effort level] was judged ‘not worth it’, they chose the ‘no’ option and the trial ended. On the other hand, if the ‘yes’ option was selected, the tree reappeared on the left or the right of the screen, indicating which hand should be used for the execution of the effortful response. The participant then had three seconds to squeeze the handgrip in order to move the red bar above the top of the trunk (Fig. 7). If the top of the trunk was not reached within the response window, no apple was gathered.

The number of apples gathered (and therefore the reward accumulated) was otherwise estimated as follow:

$$\text{Reward}=[\text{Stake}*\left(\left(\text{MaxForce}/\text{MVC})-(\text{Effort level}-0.3\right)\right)]$$

MaxForce corresponds to the maximum force reached over the 3 s response period. Participants were instructed to gather as many apples as they wanted over the course of the experiment, knowing that the money they would receive at the end would depend on the total number of apples gathered during the experiment (minimum: £8, maximum: £12). To reduce fatigue effects, blocks were interleaved with questionnaires: LARS-e, DASS and SHAPS.

The MVC for each hand was initially estimated as explained previously in the Section 2. However, to account for potential fatigue effects over time, the MVC was adjusted on each block so that it corresponded to 95% of the maximum force reached over the previous block. In addition, if a force higher than MVC was produced, the MVC would be set to this new value on subsequent trials.

#### 4.2.3 Adaptive algorithm

Importantly, the combinations of stake and effort levels on each trial were not presented at random. Instead, we used an adaptive algorithm so that the trees presented on a given trial depended on participant’s previous choices. If a combination was refused on one trial, then on a subsequent trial, the stake would increase, or the effort level would decrease (stake and effort were adjusted alternatively). The opposite would happen if a combination was accepted. We used three different staircases randomly interleaved across trials so that participants would not be aware of the procedure.

The use of an adaptive algorithm had several advantages: First, it allowed repeated sampling along subjects’ specific Indifference Points functions, which separate the stake/effort space into a ‘worth it’ and a ‘not worth it’ zone. This provided a better characterization of these functions while reducing experiment duration and fatigue. Second, it allowed for control of the number of effortful responses so that overall, every participant would accept to engage in an effortful response on ∼50% of trials.

### 4.3 Results

#### 4.3.1 Estimation of Indifference Points

**Fig. 8:**
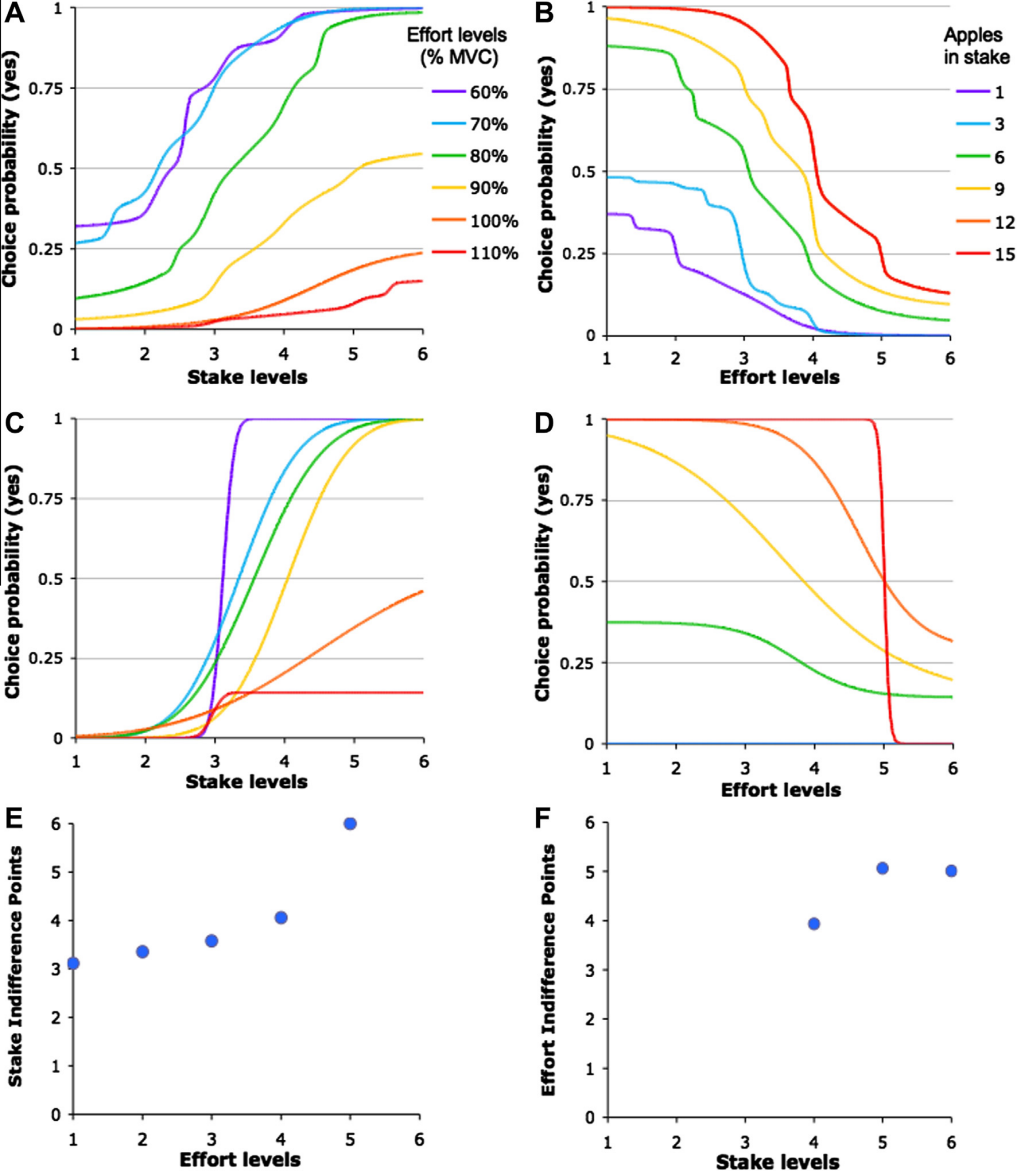
Estimation of stake and effort Indifference Points A. Probability of agreeing to engage in an effortful response (‘yes’ choice) averaged across all participants plotted as a function of stake level for each different effort levels, represented in different colors (60–110% of Maximum Voluntary Contraction (MVC) shown in different colors). (B) Probability of making a ‘yes’ choice as a function of effort levels for each different stake, represented in different colors (1–15 apples) across all participants. Panels C and D show an example from one participant of the probabilities of ‘yes’ choices, fitted with logistic functions. E. For each Effort level, plot of Stake Indifference Points corresponding to the points for which the choice probability function is equal to 0.5 (i.e. 50% chance to respond yes). (F) Similarly, Effort Indifference Points are plotted for each stake levels based on choice probability functions for the different stakes. Note that some Indifference Points cannot be estimated, as the corresponding choice probability function never reaches 0.5.

For each stake magnitude we estimated the effort level (expressed as %MVC) a subject was willing to exert on 50% of occasions, i.e. the effort Indifference Point (IP). Conversely, we also estimated for each effort level, the stake a subject was likely to accept on 50% of offers: the Stake IP. A two-step analysis was performed based on each individual’s choices. For this, we first fitted a logistic function to the choice probability data for each effort level – or each stake magnitude – separately. An example of the fitted probability functions for one subject is shown in Fig. 8C and D. The probability of agreeing to engage in a response (‘yes’ option selected) was plotted as a function of stake levels for the six different effort levels (Fig. 8C), and as a function of effort levels for the six different stakes (Fig. 8D). We also plotted the average of the fits across all participants (Fig. 8A and B). These functions then allowed estimation of effort – or stake – IPs, corresponding to the effort (or stake) value for which the probability of accepting an offer is 0.5. The IPs obtained in this way were plotted against their corresponding stakes magnitude or effort levels (Fig. 8E and F).

Overall, across the sample, increasing the stake increased the level of effort people were willing to engage to win a percentage of this stake (ANOVA main effect of stake level on Effort IP: *p* < 0.0005, Fig. 8F). Similarly, increasing effort requirements increased the stake for which participants were willing to engage (ANOVA main effect of effort level on Stake IP: *p* < 0.0005, Fig. 8E).

It could be argued that choices are not necessarily based on effort and stake, but rather on effort and potential outcome (i.e. reward that can be obtained). In our task, reward depends on performance, but it can be estimated by calculating, on each trial, the reward that could be won if participant’s maximal force were produced (i.e., if force exerted = MVC). These reward estimates were then binned into six levels to mimic the six stake levels (see Table 1).

The same procedure as explained previously was then used to estimate effort IPs for the different reward levels. As previously, when averaging the IPs across subjects, we observed a significant increase in the effort level subjects were willing to exert with increases in reward (ANOVA main effect of reward level on Effort IP: *p* < 0.0005, main effect of effort level on Reward IP: *p* < 0.0005). As would be expected, rewards for which a higher level of effort had to be produced were less desirable than when the effort requirement was low.

A limitation of this IP estimation approach is that IPs cannot always be estimated for every stake or reward level (and reciprocally), as illustrated in Fig. 8F. Indeed, for a given stake, the probability to accept responding may be consistently above or below 0.5. As a consequence, in certain subjects, only two IPs could be estimated, limiting the accurate characterization of the IP function. To estimate the characteristics of IP functions, we therefore opted for another approach consisting of directly modeling choice probabilities as a function of reward and effort, and inferring the IP function characteristics based on subject-specific parameters estimated with this model.

#### 4.3.2 Choice probability modeling

Because we were particularly interested in investigating the independent impacts of reward and effort on choice as well as the way they interact, we entered these two variables in a simple logistic regression model of choice probability:

$$P(\text{yes})={\left(1+\exp(b_{\text{rew}}*\text{reward}+b_{\text{eff}}*\text{effort}+b_{0})\right)}^{-1}$$

*P*(yes) corresponds to the probability to agree to engage in an effortful response. The *sensitivity* to reward and effort (i.e. the impact they have on choices) are reflected by the subject specific parameters given as *beta values b*_rew_ and *b*_eff_ respectively. The parameter b_0_ represent the choice bias toward the selection of the ‘no’ option, regardless of effort and reward (more bias resulting in a right shift of the sigmoid). On average across participants, reward had a weight on choice of −1.4 ± 0.6 (negative beta weight value means influence toward ‘yes’, one-sample *t*-test: *t* = −13.08, df = 29, *p* < 0.0005), effort had a weight of 0.6 ± 0.7 (*t* = 4.39, df = 29, *p* < 0.0005), and the response bias was 2.39 ± 0.60 (*t* = 3.96, df = 29, *p* < 0.0005).

#### 4.3.3 Indifference Lines characteristics

Subject-specific characteristics of the Indifference Lines (IL) separating the ‘not worth it’ from the ‘worth it’ space could be estimated based on the parameters of the choice model:

$$\text{Effort IL}=-(b_{\text{rew}}/b_{\text{eff}})*\text{reward}-b_{0}/b_{\text{eff}}$$

$$\text{Reward IL}=-(b_{\text{eff}}/b_{\text{rew}})*\text{effort}-b_{0}/b_{\text{rew}}$$

The means of these function characteristics (slope and intercept) across all participants are presented in Table 2.

The *slope* of the *Effort* IL reflects how much reward influences the subjective cost associated with effort. For instance, if the Effort IL slope is steep, this means that the subjective experience of effort is strongly associated with the reward value. Similarly, the slope of the *Reward* IL reflects the way effort requirement affects the subjective value associated with a reward. The *Effort* IL *intercept* reflects the spontaneous level of effort people are willing to engage for the smallest possible reward; while the *Reward* IL intercept corresponds to the minimal reward subjects are ready to engage in when effort requirement is minimal.

#### 4.3.4 Relation with apathy traits

Next, we examined how the model parameters related to apathy scores on the LARS-e (overall LARS-e scores and AI subscale).

##### 4.3.4.1 Apathy and sensitivity to reward or effort

No significant correlation was observed between either LARS-e or AI scores and the impact of reward or effort on choice (the beta values *b*_rew_ and *b*_eff_), or with the response bias parameter *b*_0_ (all *p* values for spearman correlations superior to 0.45). Thus apathy did not appear to be related to sensitivity to reward or effort cost across our cohort.

##### 4.3.4.2 Apathy scores are related to effort Indifference Line characteristics

**Fig. 9:**
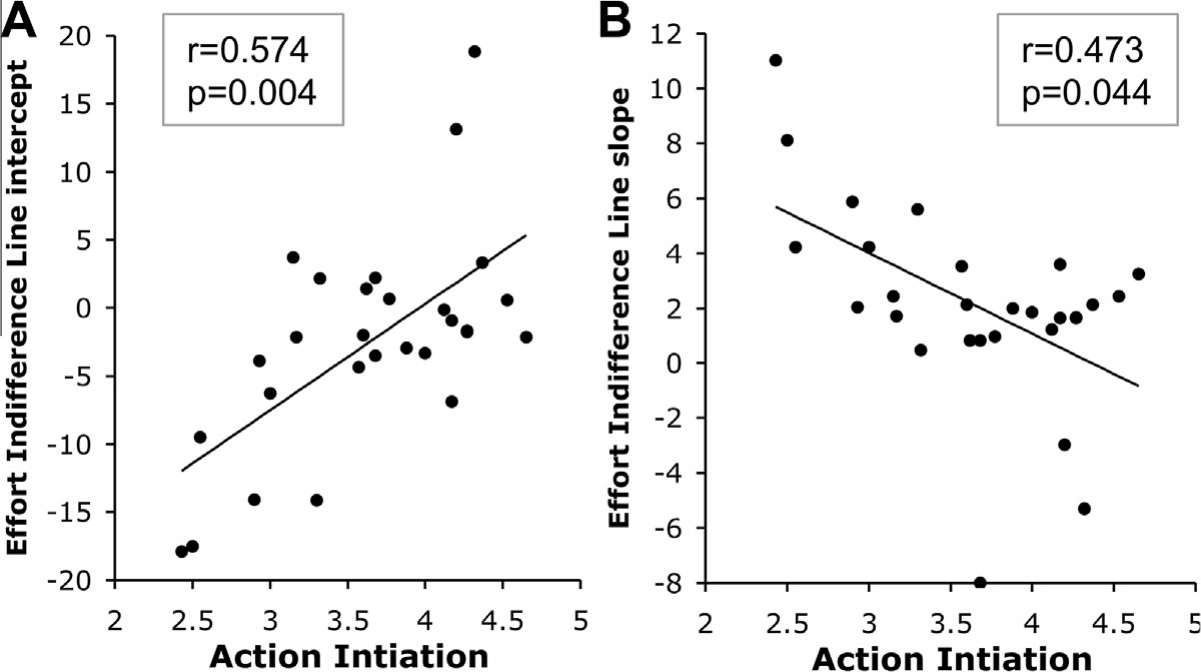
Correlations between Action Initiation scores and Effort Indifference Line characteristics. (A) Correlation with the intercept: more apathetic individuals (low AI scores) have lower intercept, which reflects a higher subjective estimation of effort cost for small rewards. (B) Correlation with the slope: more apathetic individuals have a steeper slope, which reflects a higher impact of reward on subjective effort costs.

Recall that Effort IL intercept gives the spontaneous level of effort people are willing to engage for the smallest possible reward, while Effort IL slope indexes how much reward influences the subjective cost associated with effort. LARS-e scores were significantly correlated with Effort IL *intercepts* (*r* = 0.469, *p* = 0.024; Bonferroni corrected), and more marginally with Effort IL slope (*r* = −0.385, *p* = 0.086; Bonferroni corrected). The Action Initiation (AI) component was also strongly correlated with the characteristics of the Effort IL (Correlation Ai-Effort IL intercept: *r* = 0.574, *p* = 0.004, Fig. 9A; AI-Effort IL slope, *r* = −0.473, *p* = 0.044, Fig. 9B; Bonferroni corrected).

However, intercepts and slopes were highly correlated (*r* = −0.858). We therefore examined in more detail their relationship with AI scores using partial correlations controlling for their respective variance. AI remained significantly correlated with Effort IL *intercept* while controlling for the slope (*r* = 0.419, *p* = 0.029), but the correlation between AI and Effort IL slope was no longer significant when controlling for the variance explained by the intercept (*p* = 0.51).

Importantly, the correlation between AI and Effort IL intercept remained when controlling for the other apathy subscales as well as depression and anhedonia (partial correlation *r* = 0.608, *p* = 0.002). This means that in individuals with higher apathy traits (particularly behavioral apathy) subjective effort costs for the smallest rewards were higher than in more motivated subjects (lower and negative intercept).

To further investigate how good the characteristics of the Effort IL are at classifying between high and low behavioral apathy we next divided our sample into two groups. First, we combined all the AI subscale scores of the LARS-e obtained across Experiments 1 and 2 (*N* = 80). The mean of the distribution was 3.56, with a 95% confidence interval between 3.44 and 3.69. All participants with AI scores below the lower bound of this confidence interval were classified in the ‘high apathy’ group (*N* = 12), and those with scores higher than the upper bound were classified as being in the ‘low apathy’ group (*N* = 13).

The three model parameters (*b*0_2_, *b*_rew_, *b*_eff_) and four IL characteristics for effort and reward (Effort IL slope, Effort IL intersect, Reward IL slope, Reward IL intersect) were added in a stepwise binary logistic regression analysis aimed at classifying subjects into those with high or low apathy. Only Effort IL *intercept* was selected (Chi-square = 9.12, df = 1, *p* = 0.003) in a model that could correctly classify the subjects with 80.0 % of accuracy (66.7% for the low AI group and 92.3% for the high AI group). This analysis confirms that the subjective effort cost ‘baseline’ (or intercept), which reflects how willing an individual is to engage effort when the reward is at its lowest, can be used to distinguish between subjects with high and low behavioral apathy traits.

The relationships between personality traits and Effort IL characteristics appeared to be specific to behavioral apathy, indexed by the AI subscale. There was no significant correlation with the other components of the LARS-e: Intellectual Curiosity (*p* > 0.44), Emotional Responsiveness (*p* > 0.46) and Self-Awareness (*p* > 0.46), depression (*p* > 0.5) and anhedonia (p > 0.69).

### 4.4 Conclusion

This new paradigm allows investigation of behavioral mechanisms underlying apathy traits in the healthy population. In healthy individuals with relatively high behavioral apathy traits, larger rewards are needed to initiate an effortful behavioral response. Using this measure of effort cost ‘baseline’, it was possible to classify participants into those with high or low apathy traits with 80% of accuracy, suggesting a potential for the use of this task as a tool to characterize apathy traits behaviorally, rather than with self-reports.

## 5 Discussion

In study 1, we observed that individuals with high behavioral apathy traits, as indexed by the Action Initiation (AI) subscale of the LARS-e, were more sensitive to stake and difficulty level (Figs. 4 and 5). This was mostly driven by the fact that less motivated individuals engaged less physical effort in response to least motivating conditions, but were prepared to exert similar effort as more motivated individuals for the high reward or easy conditions (Fig. 6). This could be due to differences in the absolute impact of effort level and reward on behavior (before reward/effort weighing), and/or differences in the subjective valuation of reward and effort cost relative to each other.

In order to clarify which of these behavioral mechanisms underlie apathy traits, we designed a second task to investigate physical effort and reward-based decision-making. Participants’ choices were modeled as a function of effort level and potential reward (Fig. 8). Our results suggest that apathy traits in the normal population are related to the way reward *subjectively* affects the estimation of effort costs, and more particularly manifest as decreased willingness to exert effort when rewards are small, or below threshold (smaller Effort IL intercept, Fig. 9).

Investigating apathy in a way that is not confounded by other cognitive factors is particularly critical in patients with neurological disorders, and requires a task that minimizes cognitive load, such as the one employed here. In most existing tasks designed to investigate effort discounting, participants have to choose between two options. For instance, a recent study investigating physical effort discounting used a task similar to ours, with the difference that participants have to choose between a no effort/low reward option and high efforts/high rewards options (Hartmann et al., 2013). Participants thus have to weigh reward against effort requirement in the effortful option, and then compare the subjective output of this weighing to the fixed option. In our task, participants simply had to decide whether or not they want to engage in a response given a particular combination of effort requirement and reward magnitude. This more simple approach requires less information processing, perhaps reducing potential confounds, which might be an important advantage for studying apathy in neurological or psychiatric disorders.

For the same reasons, a design such as the one we have introduced here might also be more efficient in investigating the neural basis of reward and effort-based decision-making in a neuroimaging setting, without the extra level of complexity produced by the comparison between the two options. Finally, one could argue that this type of accept/reject choice is more ecological than two-options choice. In real life, we are more often confronted by situations where we have to decide whether or not to engage in an action given a particular outcome and effort requirement, rather than situations where we have to decide between two actions with different rewards and effort requirement.

Here, we also introduced an extended version of the Lille Apathy Rating Scale (LARS-e) suitable for use in the normal population. One of the advantages of this apathy scale is that it provides separate measures of the three main apathy domains: emotional, cognitive and behavioral. Using this scale, we could therefore focus on the *behavioral domain* of apathy and control for the eventual effects related to other domains. This form of apathy is particularly frequent in patients with neurological disorders (van Reekum et al., 2005,Levy and Dubois, 2006), and is considered to be the most severe form of apathy ( Levy and Dubois, 2006). The term ‘auto-activation deficit’ has been coined to describe this syndrome, which is particularly prominent in patients with lesions of the basal ganglia, including Parkinson’s disease (Levy and Dubois, 2006).

A key challenge for future research will be to extend our task to disorders marked by prominent behavioral apathy. It may be that clinical groups exhibit a distinctly different pattern of motivational deficits (cf. Schmidt et al., 2008) to the ones we observed in the healthy population. However, our observations appear to echo some of the previously described characteristics of auto-activation deficit: behavioral inertia that is reversed by strong enough external incentives, or solicitors (Levy and Dubois, 2006).

In this study, we developed two tasks to investigate the behavioral mechanisms underlying apathy. The work we present here relates to the healthy population. It remains to be seen whether these principles might also be usefully extended to apathy in clinical populations where the syndrome is increasingly recognized to have an important functional and economic cost. The second task offers a particularly promising means to investigate further the neural basis for effort and reward based decision-making and how this may relate to individuals apathy traits.

## Figures and Tables

**Supplementary Fig. S1:**
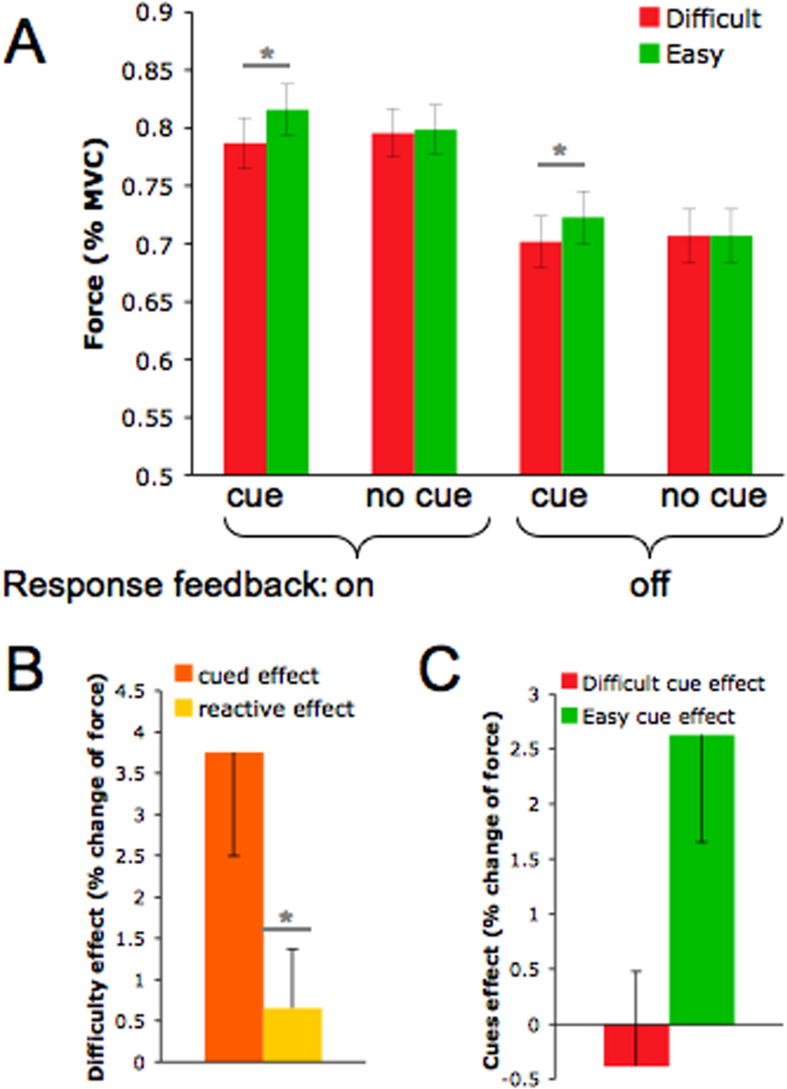
Cued and reactive effects of difficulty level on motivation. A. Force exerted, expressed as percentage of MVC for difficult and easy trials when the difficulty cue is presented or not, and when response feedback is provided or not. B. Difficulty effect expressed as percentage change of force from difficult to easy trials. Cued difficulty effect (orange) was computed by comparing the force exerted on easy and difficult trials when cues were presented but no response feedback was provided. Reactive difficulty effect (yellow) was computed by comparing the force exerted on easy and difficult trials when cues were not presented but response feedback was on. C. Effects of difficult cue (red) and easy cue (green) expressed as percentage change of force relative to no cue conditions for trials with no response feedback (±SE).

**Supplementary Fig. S2:**
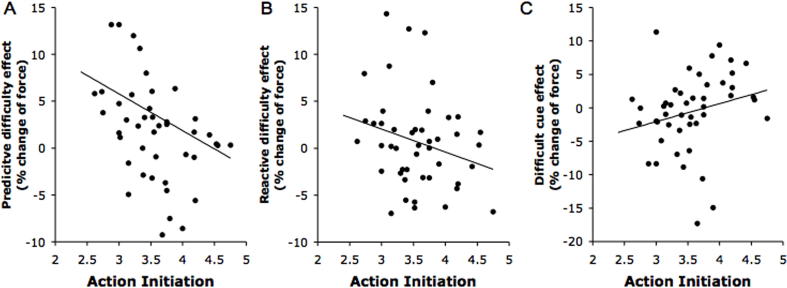
Relation between apathy traits and task performance. A. Correlation between Action Initiation subscale of LARS-e and predictive difficulty effect (percentage force change from difficult to easy cue when no response feedback provided), B. reactive difficulty effect, and C. effect of difficult cue relative to when no cue is presented.

**Table 1:** Reward estimate levels for each stake and effort combination.

Stake (apple #)	Effort levels (MVC fraction)				
	0.6	0.7	0.8	0.9	1
1	1	1	1	1	1
3	2	1	1	1	1
6	3	2	2	2	1
9	4	3	3	2	2
12	5	4	3	3	2
15	6	5	4	3	3

**Table 2:** Effort and reward Indifference Lines characteristics.

Effort IL slope (−*b*_rew_/*b*_eff2_)	Effort IL intercept (−*b*0_2_/*b*_eff2_)	Reward IL slope (−*b*_eff2_/*b*_rew_)	Reward IL intercept (−*b*0_2_/*b*_rew_)
2.14 ± 3.53	−2.60 ± 7.78	0.44 ± 0.48	0.95 ± 1.92

## Appendix A Supplementary material

Supplementary Information

Supplementary Table S1 Factor analysis of LARS-e, DASS and SHAPS scores.

AQ4-P-Self
