# Supplementary material for: Characterization of reward and effort mechanisms in apathy
Source: J Physiol Paris. 2015 Feb-Jun;109(1-3):16–26. doi: 10.1016/j.jphysparis.2014.04.002 (PMC4451957; doi:10.1016/j.jphysparis.2014.04.002)
Supplement: Supplementary Information [file mmc1.docx]

**Supplementary Information**

**1. Methods**

*1.1 Internal Consistency of the LARS-e subscales*

A reliability analysis was performed in SPSS to estimate the subscales of the LARS-e’s internal consistencies. We report here the Cronbach alpha values (a value superior to 0.7 is supposed to be acceptable):

Action Initiation: α = 0.75

Intellectual Curiosity: α = 0.81

Emotional Responsiveness: α = 0.64

Self-Awareness: α =0.52

*1.2 Relation between apathy, depression and anhedonia questionnaires*

To assess the degree to which the different apathy subscales of the LARS-e were related to each other and to depression or anhedonia, we performed a factor analysis on the questionnaires scores collected across the two studies (N=80). All LARS-e subscales were included (ER, AI, IC, SA), as well as the three subscales of the DASS (depression, anxiety and stress) and the SHAPS scores (anhedonia). This analysis was performed in SPSS, using Principal Component Analysis as the extraction method, and Varimax with Kaiser Normalization as the rotation method (Gaskin and Happell, 2013).

This analysis generated three independent components (**Table S1**). One included all the scores of the DASS as well as anhedonia scores. The second included the ER, IC and SA subscores of the LARS-e, as well as the negative anhedonia scores. The third component consisted of Action Initiation and Intellectual Curiosity from the LARS-e. The results of this analysis indicate that, in our sample, the Action Initiation subscale is partially related to Intellectual Curiosity, but importantly not to depression, anxiety, stress or anhedonia. Thus while three components of the LARS-e appear partially related to anhedonia, AI does not. As we discuss later, this might be an important dissociation to bear in mind when examining components of apathy and / or anhedonia.

**2. Results (Study 1)**

2.1. *Predictive vs. reactive influence of difficulty cues*

To examine the predictive vs. reactive modulation of motivation by the difficulty level, we next investigated the impact of difficulty cues and response feedback on effort production across all types of trials. Predictive effects occur in trials where a difficulty cue was presented; whereas reactive effects index force produced during the response when a difficulty cue had not been explicitly presented at trial onset.

An ANOVA was first performed to investigate the main effect of cue presentation and its interaction with response feedback. All the trials were included in this analysis. There was no significant effect of cue presentation (p=0.110), but there was a significant interaction cue*difficulty level (F=16.275, p<0.0005). As expected, a stronger difficulty effect was observed when the difficulty cues were presented (**Figure S1A**). There was no other significant interaction with difficulty cue presentation.

We then looked in more details at the predictive and reactive effect of difficulty cue. The predictive effect of the difficulty cue (cue presented but no response feedback) was significant (one-sample t-test, t=3.335, df=49, p=0.002). However, the reactive effect (cue not present but response feedback provided) was not (**Figure S1B**). The predictive cue effect was significantly greater than the reactive one (paired t-test, t=2.513, df=49 p=0.015). This result indicates that motivation is more strongly modulated *before* the response by the presentation of difficulty cues than on-line during experience of the difficulty level as participants exerted force.

2.2. *Stronger impact of ‘Easy’ cue relative to ‘Difficult’ cue on motivation*

We next examined the ‘facilitatory’ and ‘undermining’ effects of the easy and difficult cues. To compute the facilitatory effect of the easy cue, we compared the response force between ‘easy’ trials with response feedback off when the easy cue was presented vs. easy trials on which no cue and no response feedback was provided. The presentation of easy cues provoked a significant increase in response force (one-sample t-test, t=3.15, df=49, p=0.003, **Figure S1C**), but the difficult cue did not provoke an overall significant undermining effect. The facilitatory effect of the easy cue was stronger than the undermining effect of the difficult cue (t=2.079, df=49, p=0.043). Thus, on average, participants were more motivated by the presentation of a cue indicating the trial would be easy than they were demotivated by a cue indicating the trial would be hard.

2.3. Relation between behavioural apathy and difficulty cues effects

We investigated the effects of difficulty levels in more detail. As mentioned above, in this task, difficulty levels can modulate motivation via two different processes. One is predictive, assessed by comparing the effort exerted when a difficulty cue is presented or not in the absence of response feedback. The other is reactive, measured by comparing the effort exerted with and without response feedback when no difficulty cue is presented. Both predictive (r=-0.379, p=0.007) and reactive (r=-0.309, p=0.029) modulations of effort production by difficulty level (Easy vs. difficult) were significantly correlated with LARS-e scores.

Interestingly, only the predictive modulation type was significantly correlated with the Action Initiation subscale (r=-0.406, p=0.003, **Figure S2A**), not the reactive modulation (p=, **Figure S2B**). The other subscales were not significantly correlated with these effects. This suggests that individuals with more behavioural apathy are *more sensitive* to cued difficulty during response preparation, than after a response has been initiated.

We previously observed that the ‘Difficult’ and the ‘Easy’ cues have different impact on motivation. There is an undermining effect of the presentation of the difficult cue and a facilitatory effect of the easy cue, compared to when there is no information about difficulty level (no cue presented). Interestingly, only the undermining effect of the ‘Difficult’ cue was significantly related to the Action Initiation subscale of the LARS-e (r=0.351, p=0.018) (**Figure S2C**). Individuals with higher apathy traits (especially behavioral) were more subjects to the undermining effect of the difficult cue. By contrast, among the more motivated subjects, some even showed a positive, perhaps stimulating effect of the ‘Difficult’ cue on effort production.

**Supplementary tables and figures legends**

**Table S1: Factor analysis of LARS-e, DASS and SHAPS scores**

Factor Loadings for each of the questionnaire subscales. Emotion, Action Initiation, Intellectual Curiosity and Self-Awareness are the four subscales of the LARS-e. Depression, Anxiety and Stress are the three subscales of the DASS. Anhedonia refers to the SHAPs score.

**Figure S1:** **Cued and reactive effects of difficulty level on motivation.** **A.** Force exerted, expressed as percentage of MVC for difficult and easy trials when the difficulty cue is presented or not, and when response feedback is provided or not. **B.** Difficulty effect expressed as percentage change of force from difficult to easy trials. *Cued difficulty effect* (orange) was computed by comparing the force exerted on easy and difficult trials when cues were presented but no response feedback was provided. *Reactive difficulty effect* (yellow) was computed by comparing the force exerted on easy and difficult trials when cues were not presented but response feedback was on. **C.** Effects of difficult cue (red) and easy cue (green) expressed as percentage change of force relative to no cue conditions for trials with no response feedback (± SE).

**Figure S2: Relation between apathy traits and task performance.**

**A.** Correlation between Action Initiation subscale of LARS-e and predictive difficulty effect (percentage force change from difficult to easy cue when no response feedback provided), **B.** reactive difficulty effect, and **C.** Effect of difficult cue relative to when no cue is presented.
