# Supplementary material for: Characterization of reward and effort mechanisms in apathy
Source: J Physiol Paris. 2015 Feb-Jun;109(1-3):16–26. doi: 10.1016/j.jphysparis.2014.04.002 (PMC4451957; doi:10.1016/j.jphysparis.2014.04.002)
Supplement: Supplementary Table S1 — Factor analysis of LARS-e, DASS and SHAPS scores. [file mmc2.doc]

**Table S**1: Factor analysis of LARS-e, DASS and SHAPS scores

|  | **Components^a^** | | |
| --- | --- | --- | --- |
|  | **1** | **2** | **3** |
| Emotion |  | .836 |  |
| Action Initiation |  |  | .925 |
| Intellectual Curiosity |  | .432 | .652 |
| Self-Awareness |  | .747 |  |
| Depression | .931 |  |  |
| Anxiety | .922 |  |  |
| Stress | .907 |  |  |
| Anhedonia | .456 | -.586 |  |

^a^Rotated Component Matrix output of the factor analysis performed in SPSS using Principal component analysis as extraction method, and varimax as rotation method. Rotation converged in 4 iterations.
