# Supplementary material for: Characterization of reward and effort mechanisms in apathy
Source: J Physiol Paris. 2015 Feb-Jun;109(1-3):16–26. doi: 10.1016/j.jphysparis.2014.04.002 (PMC4451957; doi:10.1016/j.jphysparis.2014.04.002)
Supplement: AQ4-P-Self [file mmc3.pdf]

Date __16-01-13____________
ID ______________

AQ4-P-Self

Below are a number of statements. Each statement asks you to think about your life over the last 2 weeks.

For each statement, circle a number 1-5 to indicate how true the statement is of you over the last 2 weeks, i.e. how appropriately it describes your life right now.

Circle “1” if the statement does not describe you at all over the last 2 weeks, “5” if the statement describes you perfectly, and use the numbers in between accordingly. Circle “NA” if the statement is not applicable to you over the last 2 weeks.

1 = Completely UNTRUE: The statement is exactly the opposite of me over the last 2 weeks
2 = Mostly untrue of me over the last 2 weeks
3 = Neither untrue nor true of me over the last 2 weeks
4 = Quite true of me over the last 2 weeks
5 = Completely TRUE: The statement exactly describes me over the last 2 weeks

	**In the last 2 weeks…**	UN-TRUE		=		TRUE	
1<br>E-ER	I felt bad when I heard an acquaintance had had an accident or illness.	1	2	3	4	5	NA
2<br>AI-EP	I didn’t like to laze around.	1	2	3	4	5	NA
3<br>IC-N	If a new restaurant opened locally, I'd want to try it out.	1	2	3	4	5	NA
4<br>SA	If I realised I'd been unpleasant to someone, I felt terribly guilty afterwards.	1	2	3	4	5	NA
5<br>AI-EP	I liked to have a busy schedule.	1	2	3	4	5	NA
6<br>IC-S	I started conversations with random people.	1	2	3	4	5	NA
7<br>AI-EP	I usually got up in the morning even if I didn't have to go to work.	1	2	3	4	5	NA
8<br>IC-M	When I didn’t manage to do something, I tried to find other solutions.	1	2	3	4	5	NA
9<br>IC-S	I enjoyed spending time with my friends.	1	2	3	4	5	NA
10<br>SA	If I couldn't do something, I accepted help from others.	1	2	3	4	5	NA
11<br>AI-I	I got things done when they need to be done, without being reminded by others.	1	2	3	4	5	NA
12<br>AI-I	I made decisions firmly and without hesitation.	1	2	3	4	5	NA
13<br>IC-N	If someone had asked me “Would you like to visit every continent in the world?” I’d have said yes.	1	2	3	4	5	NA
14<br>AI-I	When I had something I needed to do, I did it straightaway so it was out of the way.	1	2	3	4	5	NA

	In the last 2 weeks…	UN-TRUE = TRUE					
15<br>SA	I changed my mind during an argument when I realised the other person might be right.	1	2	3	4	5	NA
16<br>AI-I	I liked being in charge.	1	2	3	4	5	NA
17<br>IC-M	If I had made a list of my attributes, my ability to push myself to achieve would have been one of them.	1	2	3	4	5	NA
18<br>E-ER	I laughed out loud when listening to a comedy show.	1	2	3	4	5	NA
19<br>IC-N	I enjoyed visiting new places.	1	2	3	4	5	NA
20<br>IC-M	When I couldn't find something (e.g. a document), I went to a lot of trouble looking for it.	1	2	3	4	5	NA
21<br>SA	Based on the last 2 weeks, I'd say I care deeply how my loved ones think of me.	1	2	3	4	5	NA
22<br>AI-EP	If I didn't achieve my goals for the day, I got annoyed.	1	2	3	4	5	NA
23<br>E-ER	I felt sad or upset when I heard bad news.	1	2	3	4	5	NA
24<br>AI-I	If I had to RSVP to an invitation, I did it promptly, without needing a reminder.	1	2	3	4	5	NA
25<br>IC-S	I suggested activities for me and my friends to do.	1	2	3	4	5	NA
26<br>IC-I	I enjoyed choosing what to do from a range of activities.	1	2	3	4	5	NA
27<br>IC-N	I enjoyed doing things with people I'd just met.	1	2	3	4	5	NA
28<br>AI-I	I was easily able to decide to do things by myself, without needing someone to push or encourage me.	1	2	3	4	5	NA
29<br>IC-S	I started conversations without being prompted.	1	2	3	4	5	NA
30<br>E-ER	When I listened to music, it could affect me deeply.	1	2	3	4	5	NA
31<br>IC-N	I enjoyed trying out products, tools or recipes that I was unfamiliar with.	1	2	3	4	5	NA
32<br>SA	After having taken a decision, I wondered if I 'd made the wrong choice.	1	2	3	4	5	NA
33<br>IC-M	When I decided to do something, I was motivated to see it through to the end.	1	2	3	4	5	NA
34<br>IC-S	If I hadn't seen a close friend for a while, I got in touch by phone/internet.	1	2	3	4	5	NA
35<br>SA	When I finished doing something, I took stock of the situation to identify what was going well and what wasn't.	1	2	3	4	5	NA
36<br>IC-N	I enjoyed learning new things at work.	1	2	3	4	5	NA
37<br>IC-S	I was pleased when I received unexpected invitations from friends.	1	2	3	4	5	NA
38<br>IC-I	I would say I've been quite interested in life.	1	2	3	4	5	NA

	In the last 2 weeks…	UN- TRUE		=		TRUE	
39 IC-M	When I decided to do something, I was easily able to make an effort.	1	2	3	4	5	NA
40 SA	I felt awful if I said something insensitive.	1	2	3	4	5	NA
41 IC-I	I've been feeling there is so much to do in life and I want to try everything.	1	2	3	4	5	NA
42 E-ER	When someone told me a joke I laughed easily.	1	2	3	4	5	NA
43 AI-EP	Getting something done by 9am gave me a sense of satisfaction.	1	2	3	4	5	NA
44 SA	If, during a discussion, I realised I was in the wrong, I admitted it – at least to myself.	1	2	3	4	5	NA
45 IC-S	I've been going out with friends on a weekly basis.	1	2	3	4	5	NA
46 IC-M	I found it annoying when I was in the middle of doing a task at work and I had to stop to do something else.	1	2	3	4	5	NA
47 IC-I	I wished I had more spare time for hobbies and leisure activities.	1	2	3	4	5	NA
48 IC-M	If I thought of something I needed to do, I wrote it down so I wouldn't forget.	1	2	3	4	5	NA
49 IC-M	I tried out new ways of doing things at work or home.	1	2	3	4	5	NA
50 E-ER	I felt anxious when I was waiting to hear important news (e.g. exam result or job interview).	1	2	3	4	5	NA
51 E-ER	I was easily 'moved' by films/TV programs.	1	2	3	4	5	NA
